# Perfusion CT and PET with 18F–FDG and 18F–FCh in the complex diagnosis of hepatocellular carcinoma

**DOI:** 10.1186/s41824-017-0018-7

**Published:** 2017-12-01

**Authors:** P. E. Tulin, M. B. Dolgushin, A. A. Odzharova, A. I. Mikhaylov, B. M. Medvedeva, S. V. Shiryaev, B. I. Dolgushin

**Affiliations:** 1 Department of positron emission tomography of N.N. Blokhin Russian Cancer Research Center, Kashirskoe shosse, 23, Moscow, 115478 Russia; 2 ICERR FSBSI N.N. Blokhin RCRC, Moscow, Russia; 3 Department of nuclear medicine and radionuclide therapy of N.N. Blokhin RCRC, Moscow, Russia

**Keywords:** 18F–FDG, 18F–FCh, CT-perfusion, Hepatocellular carcinoma

## Abstract

**Background:**

The purpose of the study to evaluate possibilities of CT-perfusion and PET methods with 18F–FDG and 18F–fluorocholine in the complex diagnosis of hepatocellular carcinoma. The study included the results of PET/CT with 18F–FDG, 18F–FCh and CT-perfusion of the liver in 18 patients with histologically confirmed diagnosis of hepatocellular carcinoma (HCC). Depending on the degree of tumor differentiation, all patients were divided into 3 groups - patients with highly differentiated (6 patients), moderately differentiated (4 patients), and low-differentiated HCC (8 patients).

**Results:**

Average values of maxSUV in the group of patients with highly differentiated HCC in PET/CT with 18F–FDG and 18F- fluorocholine in a solid component of tumor reached 3.51 and 18.24, respectively; in patients with moderately differentiated HCC - 3.91 and 12.32, respectively; in patients with low-differentiated HCC - 9.58 and 9.70, respectively. Average values of CT perfusion imaging in a solid component of the tumor in the group of patients with highly differentiated HCC were the following: BF - 55,33 ml/100 ml/min, BV - 13,71 ml/100 ml, ALP - 52,41 ml/100 ml/min, PVP - 10.81 ml/100 ml/min (*p* ≤ 0,05), in the group of patients with moderately differentiated HCC: BF - 52,78 ml/100 m /min, BV - 12,23 ml/100 ml, ALP - 47,26 ml/100 ml/min, PVP - 9,10 ml/100 ml/min (*p* ≤ 0.05), in the solid component of low-differentiated HCC: BF - 46,96 ml/100 ml/min, BV - 9,49 ml/100 ml, ALP - 40.54 ml/100 ml/min, PVP - 7,66 ml/100 ml/min (*p* ≤ 0,05).

**Conclusions:**

The diagnostic capabilities of the complex of PET/CT techniques with 18F–FDG and 18F–FCh and CT perfusion in a single-scan mode for hepatocellular carcinoma were evaluated for the first time. The obtained data allow to assume that the integrated use of PET with 18F–FDG and 18F–FCh and CT perfusion in a single scan improves the differential diagnostic possibilities of PET/CT diagnostics, which can find application in planning and prognosis of the disease. Due to the small number of patients further study of the problem is required.

## Background

Primary liver tumors account for 0.7% of all forms of cancer. Liver cancer is the fifth most common among men (7.5%) and the ninth - among women (3.4%) (GLOBOCAN, 2014; Bray et al., 2012). High death rate and permanently growing incidence of malignant liver tumors require improvement of methods of diagnosis and treatment.

Hepatocellular carcinoma (HCC) is a malignant tumor of the liver that develops from hepatocytes. The share of hepatocellular cancer accounts for 80–90% of primary malignant tumors of the liver (Patyutko Yu et al., 2011). Men get sick 2 times more often - the average age of patients is 50 years (Chissov, 2008). Mortality in a year after the detection of the disease in men is 75%, in women - 71.4% (Sukonko, 2012). In 60–90% of cases, HCC occurs against the background of liver cirrhosis, a common cause of which is hepatitis B or C (Podymova, 1998; Bosch et al., 1998; Beasley et al., 1981), with the prevalence of HCV infection (Highleyman, 2015; Miller et al., 2015; Soget et al., 2013). There are six main histological forms of HCC growth: trabecular, pseudo-ferrous, cirrhotic, solid, fibrolamellar, spindle cell (Salomao et al., 2012). By the degree of cell differentiation HCC are divided on high-differentiated, moderately differentiated, low- and undifferentiated types. (Ershov, 2009).

The main methods for noninvasive diagnosis of HCC currently are: ultrasonography (US), computed tomography (CT) and magnetic resonance imaging (MRI). Direct angiography and positron emission tomography (PET) are used less often.

US, CT and MRI with the introduction of contrast agents have different specificities in determining the nature of liver tumors (Matsui et al., 1991; Bartolotta et al., 2009; Tiferes & D’ippolito, 2008; Mirk et al., 1982; Ternovoy & Shiganova, 1999; Lukyanchenko & Medvedev, 2004; van den Esschert et al., 2011; Talbot et al., 2010; Delbeke et al., 1998; Tulin et al., 2015; Karmazanovskii & Szymanowski, 2007). Wide-scale application of tissue-specific magnetic resonance contrast substances based on gadoxetic acid, which are selectively absorbed by hepatocytes, greatly increases the diagnostic value of MRI examination in HCC (Pantoja, 1968).

Different histological types of liver neoplasms characterized by the distinctive features of the vascular architecture (Zajko et al., 1985; Dolgushin et al., 2012). However, evaluation of the tissue and cellular matrix of the tumor, determination of the true border of its spread, the degree of tumor vascularization and its angiogenesis are possible today only with the help of molecular imaging methods (Meier & Zierler, 1954). One of the methods for quantifying the hemodynamic properties of tissues is perfusion. For the first time, the perfusion method was applied to the evaluation of parameters of cerebral blood flow (speed (CBF) and volume (CBV)) using intravascular contrast agent (CA) in the early 50-ies of XX century (Kety & Schmidt, 1948; Pronin et al., 2007).

In 1980, a technique for evaluating the perfusion of the brain with the help of dynamic CT was proposed. By recording changes in the density characteristics of the tissue as the iodine-based contrast media passes through its vasculature (Axel, 1980; Mathieu et al., 1986). The method allowed quantitatively studying hemodynamic parameters of tumor tissue and surrounding anatomical structures. The first results of CT perfusion (CTP) of the liver appeared in the late 80’s and early 90’s of the twentieth century, but the weak possibilities of mathematical data processing and the low resolving power of tomographs limited their use for this purpose (Miles, 1991; Rees, 1992; Miles et al., 1993; Kuang, 2009).

Another method of molecular imaging that can assess the molecular and cellular tumor matrix is PET. The method of positron emission tomography reflects the metabolism of a tumor cell. Cells of hepatocellular carcinoma, depending on the degree of differentiation, have different enzymatic activity (Ershov, 2009; Pritchard & Vance, 1981; Yamamoto et al., 2008; Paudya et al., 2008), therefore, using radiopharmaceuticals agents aimed at visualization and evaluation of the main chains of hepatocyte metabolism - 18F-fluorodeoxyglucose (18F–FDG) and 18F–fluorocholine (18F–FCh), it is possible to carry out differential diagnosis of HCC in terms of the degree of differentiation (Talbot et al., 2010; Hwang et al., 2009; Nordlie et al., 1999; Berteloot et al., 1991).

In this study, the diagnostic capabilities of the complex of PET/CT techniques with 18F–FDG and 18F–FCh and CT perfusion in a single-scan mode for hepatocellular carcinoma were evaluated for the first time. The main objective of the study is to compare the results of positron emission tomography (using 18F–FDG and 18F–FCh) and CT perfusion in hepatocellular carcinoma of various degrees of differentiation.

## Methods

The study included data from CT perfusion and PET / CT with 18F–FDG and 18F–FCh, performed in 18 patients with hepatocellular carcinoma. Depending on the degree of differentiation of tumors, all patients were divided into 3 groups - patients with highly differentiated (6 patients), moderately differentiated (4 patients) and low-differentiated (8 patients) HCC. All patients underwent fine needle aspiration and surgical removal of neoplasms in the liver followed by histological examination of the material. In 3 patients hepatitis C was detected, in 6 patients - hepatitis B. Liver cirrhosis was detected in 7 patients (6 of them had hepatitis) with the severity of the current corresponding to Child A class Child-Pugh classification. Until inclusion in the study patients did not receive special treatment.

Before the PET/CT examination, each patient underwent CT scan of the abdomen with intravenous administration of 100 ml «Omnipaque» (in the three-phase scanning mode), 12 patients underwent MRI in the modes: T1-and T2-weighted images, DWI (diffusion-weighted image, b = 50, 600, 800 s/mm^2^). According to the results of CT and MRI, only the right lobe of the liver was affected in 9 patients, in 4 patients only the left lobe of the liver, lesions in both liver lobes were detected in 5 patients.

Each patient underwent sequentially PET/CT with 18F–FDG, PET/CT with 18F- FCh and CT perfusion of the liver (in a single-scan mode).

### PET/CT with 18F–FDG (Siemens biograph mCT)

The studies were performed on an empty stomach (at least 6 h of fasting) with a water load (0.5 l of water). 308–501 MBq 18F–FDG was administered intravenously (depending on the weight of the patient). Scanning was performed after 60 min. The duration of each 18F–FDG PET study was 3 min /bed. On a series of obtained tomographic images, foci of pathological accumulation of the radiopharmaceutical agent in solid areas of the tumor were identified. The levels of accumulation of the radiopharmaceutical agent (maxSUV - standardized uptake value) in the solid areas of tumor sites, in areas of necrosis and in the unchanged liver parenchyma were measured.

### PET/CT with 18F–FCh (Siemens biograph mCT)

Scaning was performed after 40 min of 310–501 MBq 18F–FCh intravenous administration, the levels of radiopharmaceutical agent accumulation in the solid areas of the tumors, areas of necrosis, the unchanged liver parenchyma were measured.

For all patients CTP was performed on a Siemens Biograph mCT scanner in a single-scan mode with PET/CT to maintain the patient’s most similar vital parameters during the study (without changing the position of the patient’s body after a PET/CT scan) using the following specifications (Table 1).

**Table 1:** Technical parameters of CT perfusion of the liver

KVp	100
mAs	150
The amount of “Omnipaque” 300 mg/ml with intravenous injection	50 ml
The rate of infusion of “Omnipaque”	2,5–4 ml/s
Concentration of “Omnipaque”	300 mg/ml
Scan delay	8 s
Rotation time of X-ray tube	1 s
Total scan time	45 s
Slice thickness	2,0 mm
Pitch	0

A CA was injected in a cubital vein in the patient’s hands behind his head. Previously each patient was given instruction on the method of performing the research (“rehearsal” activity and depth of breathing movements) to minimize artifacts from the excursions of the diaphragm. An elastic retainer was applied to the region of the chest, diaphragm and the upper abdominal wall of the patient to limit movement activity during breathing.

**Fig. 1:**
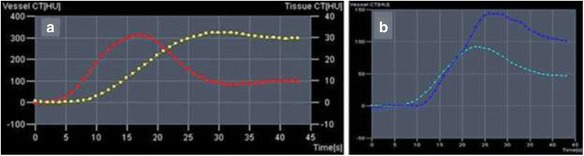
Graphs of “concentration/time”. **a** Values of arterial blood flow (red curve) ROI - abdominal aorta, surrounding tissue (yellow curve). **b** Indicators of blood flow in the stroma of the spleen (dark blue curve) and portal vein (blue curve)

After receiving a series of CTP images, data processing was performed at the Siemens Multy Modality Workplace workstation in off-line mode. The image quality was adjusted for breathing and noise of the heterogeneity of tissue density parameters. For the quantitative analysis, the basic anatomical areas for the calculation of the perfusion parameters were recorded - aorta (the region is determined automatically with the formation of the concentration/time curves at threshold from −50 HU to +60 HU), portal vein, spleen and several areas of interest in the tumor tissues (areas are selected manually). Peak contrast enhancement (the highest values of Hounsfield’s units) in the aorta was achieved in 17 s (±2 s) (Fig. 1). Peak contrast enhancement of the portal vein for 30 s (±4 s), peak contrast enhancement of the spleen revealed a few previously – 20-25 s (regardless of age and physique of the patient).

Quantitative analysis was performed on the following parameters: BV (blood volume in the total mass of the tumor: ml/100 ml), BF (hepatic blood flow: ml/100 ml/min), ALP (liver arterial perfusion: ml/100 ml/min), PVP (liver portal perfusion: ml/100 ml/min). In the tumor structure, on color parametric maps, the indicators were measured in the following areas of interest: a solid tumor component, a necrosis area and cystic cavities, if any were available. To correct the obtained data and to determine miscalculations of the performed scanning, the perfusion parameters in the unaffected parenchyma of the liver and in the spleen were further analyzed. The time interval for scanning did not exceed 45 s.

## Results

The average values of 18F–FDG accumulation in solid sections of nodes of low differentiated - HCC were maxSUV 9.58, in the necrosis zones - maxSUV 1.01, in the unchanged parenchyma - maxSUV 2.61; in the solid sections of the nodes of the highly differentiated HCC - maxSUV 3.51, in the necrosis zones - maxSUV 0.89, in the unchanged parenchyma - maxSUV 2.53; in the solid sites of moderately differentiated HCC nodes - maxSUV 3.91, in the zones of necrosis - maxSUV 1.00, in the unchanged parenchyma maxSUV 2.14 (*p* ≤ 0.05).

**Fig. 2:**
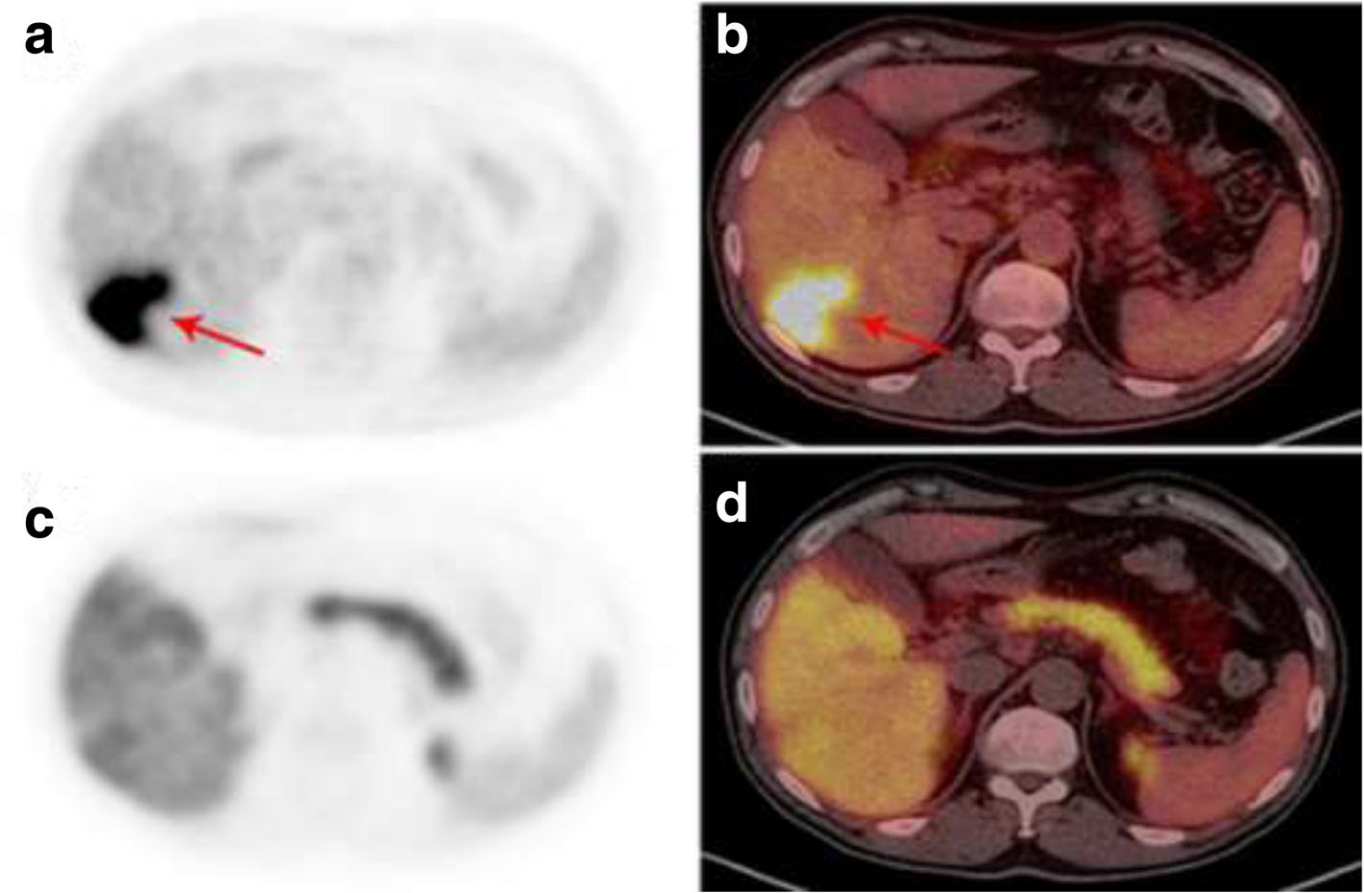
PET/CT with 18F–FDG and 18F–FCh in low-differentiated hepatocellular carcinoma. Patient (**a**). Low-differentiated hepatocellular carcinoma. Images of axial projections of PET (**a**, **c**) and combined images of PET/CT (**b**, **d**) with 18F–FDG (**a**, **b**) and with 18F–FCh (**c**, **d**). With PET/CT with 18F–FDG in the right lobe of the liver, the focus of pathological accumulation of the radiopharmaceutical agent (red arrows) is visualized. With PET/CT with 18F–FCh, the pathological accumulation of 18F–FCh in the liver is not determined

The average values of 18F–FCh accumulation in solid sections of nodes of low differentiated HCC were maxSUV 7.07, in the zones of necrosis - maxSUV 0.76, in the unchanged parenchyma - maxSUV 9.70; in the solid sections of nodes of highly differentiated HCC - maxSUV 18.24, in the zones of necrosis - maxSUV 0.81, in the unchanged parenchyma maxSUV - 8.58; in the solid sites of moderately differentiated HCC nodes - maxSUV 12.32, in the zones of necrosis - maxSUV 0.94, in the unchanged parenchyma - maxSUV 10.45 (*p* ≤ 0.05) (Table 2) (Fig. 2).

**Table 2:** Average values of accumulation of 18F–FDG and 18F–FCh in tumor sites and in the normal hepatic parenchyma

	^18^F–FDG	^18^F–FCh
The average maxSUV in unaltered liver parenchyma in all patients	2,42	9,57
The average maxSUV in the area of highly differentiated HCC	3,51	18,24
The average maxSUV in the area of low-differentiated HCC	9,58	9,70
The average maxSUV in the area of moderately differentiated HCC	3,91	12,32

**Fig. 3:**
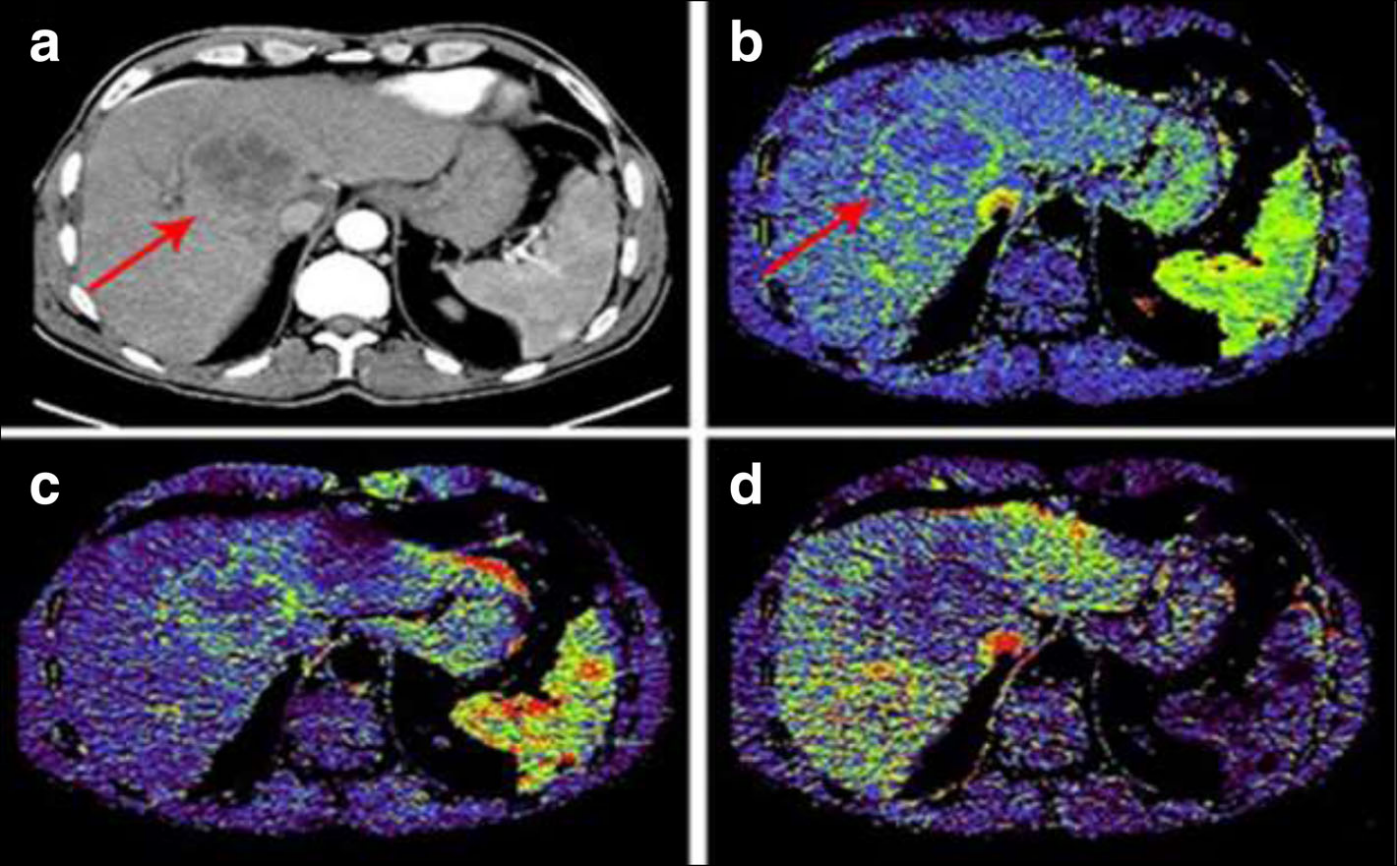
CT-perfusion in moderately differentiated hepatocellular carcinoma. Patient P. Moderately differentiated hepatocellular carcinoma. Images of axial sections of CT with intravenous contrast (arterial phase) (**a**), perfusion maps BV (**b**), ALP (**c**), PVP (**d**). Nodal formation in the right lobe of the liver (red arrows) unevenly accumulates CA (**a**). According to the CTP data, an increase in the volume of arterial blood flow along the periphery of the tumor (**b**, **c**) is detected, a decrease in portal blood flow (**d**)

**Fig. 4:**
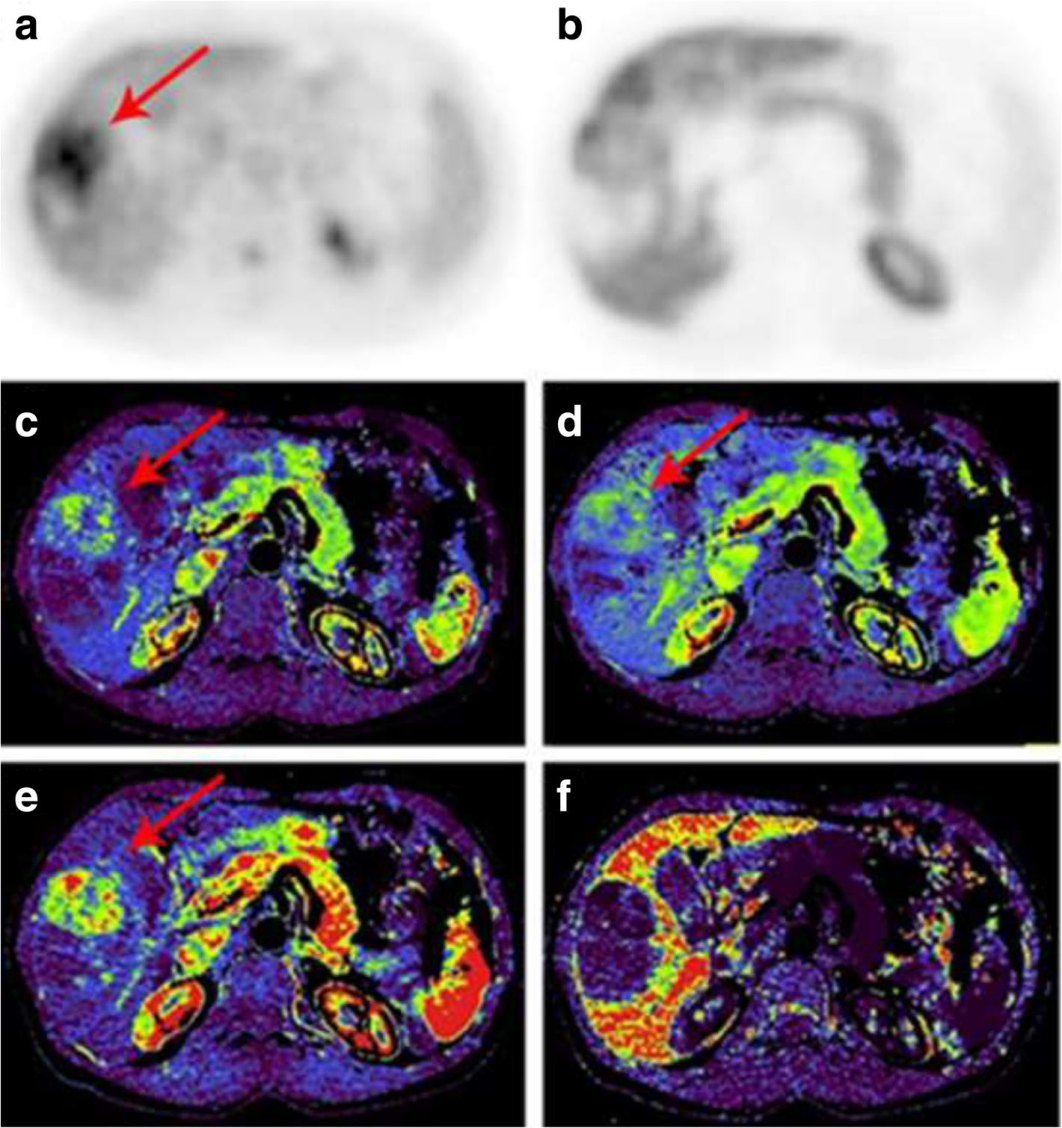
PET/CT and CT-perfusion in low-differentiated hepatocellular carcinoma. Patient B. Low-differentiated hepatocellular carcinoma. Images of axial slices of PET with 18F–FDG (**a**), 18F–FCh (**b**), BF perfusion maps (**c**), BV (**d**), ALP (**e**), PVP (**f**). Nodal formation in the right lobe of the liver (arrows) accumulates 18F–FDG (**a**), does not accumulate 18F–FCh (**b**). Perfusion maps visualize increased intensity and volume of arterial blood flow in the tumor (**c**, **d**, **e**), decrease of the portal component (**f**)

The quantitative analysis showed the average perfusion values in the solid component of the highly differentiated HCC: BF 55.33 ml/100 ml/min, BV 13.71 ml/100 ml, ALP 52.41 ml/100 ml/min, PVP - 10.81 ml/100 ml/min; in the solid component of the low differentiated HCC: BF - 46.96 ml/100 ml/min, BV - 9.49 ml/100 ml, ALP - 40.54 ml/100 ml/min, PVP - 7.66 ml/100 ml/min; in the solid component of moderately differentiated HCC: BF - 52.78 ml/100 ml/min, BV - 12.23 ml/100 ml, ALP - 47.26 ml/100 ml/min, PVP - 9.10 ml/100 ml/min (*p* ≤ 0.05) (Table 3) (Figs. 3 and 4).

**Table 3:** Perfusion mean values in tumor nodes and in normal hepatic parenchyma

	BF (ml/100 ml/min)	BV (ml /100 ml)	ALP (ml /100 ml/min)	PVP (ml/100 ml/min)
Unchanged parenchyma	29,22	11,75	21,06	90,44
Highly differentiated HCC	55,33	13,71	52,41	10,81
low-differentiated HCC	46,96	9,49	40,54	7,66
Moderately differentiated HCC	52,78	12,23	47,26	9,10

There are signs of high vascularization of HCC nodes with a predominance of arterial perfusion (high values of BF and ALP), while blood volume (BV) and portal blood flow (PVP) are reduced in them.

The values of CT perfusion in the solid portions of the moderately differentiated HCC occupied an intermediate position between the highly and low-differentiated forms. The parameters of portal perfusion (PVP) were significantly reduced in all types of cancer differentiation in relation to the unchanged liver parenchyma, but they did not differ significantly with each other.

There were no statistically significant deviations in perfusion of the unaffected by tumor liver tissue in patients with and without cirrhosis.

## Discussion

Cells of hepatocellular cancer are characterized not only by altered structure, but also by pathological metabolism. Biochemical processes, namely the sequence of anabolism and catabolism, are controlled by enzymes, however, their concentration (as in the extracellular fluid and the cytoplasm) varies considerably depending on cancers differentiation degree, therefore, critical for hepatocyte processes, such as glucose and choline metabolism, occur in different ways are processes like. Normally, glucose transport into the cell takes place through glucose transporter GLUT-2. Inside the cell, glucose is phosphorylated by the enzyme type 2 hexokinase to glucose-6-phosphate. The basic final substances of glucose metabolism chains in hepatocytes are glycogen, pentoses, and fatty acids. The dephosphorylation reaction is catalyzed by glucose-6-phosphatase. There is a significant decrease in glucose-6-phosphatase, a high concentration of GLUT-2 and membrane receptors with which the enzyme-transporter interacts in the cell of the low- differentiated HCC. Consequently, glucose molecules in a larger amount enter the cell and undergo phosphorylation – it’s displayed by 18F–FDG PET/CT as focal pathological accumulation of the radiopharmaceutical. In the cell of the highly differentiated hepatocellular cancer, the glucose-6-phosphatase concentration is higher than in the unchanged hepatocyte, and the number of GLUT-2 molecules is reduced, which prevents glucose molecules from integrating into metabolic cascades in the volume required for imaging with 18F –FDG PET/CT (Argaud et al., 1997; Zeisel & Blusztajn, 1994; Ackerstaff et al., 2003).

Choline, as part of the phosphatidylcholine molecule, is a major structural component of cell membrane of all cells. The molecule of choline is transported inside the cell by a transmembrane transporter - cholintransferase. The mechanism of phosphatidylcholine formation is fundamental for hepatocyte membrane build. Within the cell, the choline molecule undergoes phosphorylation with the formation of phosphocholine with the help of the enzyme-catalyst choline kinase. Phosphocholine through a series of cascade reactions is transformed into a molecule of phosphatidylcholine. The dephosphorylation process (phosphocholine-choline) catalyzes by choline phosphatase. An alternative transformation way of the choline molecule into phosphatidylcholine is a series of oxidative reactions with the formation of betaine and methionine. In the cell of highly differentiated HCC an increased concentration of choline kinase and active oxidative processes are noted, which leads to an intensive pathological metabolism of choline, which is reflected in 18F- FCh PET/CT as hypermetabolic foci of radiopharmaceutical accumulation. Other processes are noted in the cell of a low differentiated HCC - a low concentration of choline kinase and a high concentration of choline phosphatase lead to a marked decrease in lipid metabolism. Thus, 18F–FCh PET/CT is not informative in patients with a low differentiated HCC (Miles, 1991; Pritchard & Vance, 1981; Yamamoto et al., 2008; Ackerstaff et al., 2003).

The peculiarities of the interaction between the portal and arterial bloodstream of the liver were investigated in the middle of the twentieth century (Ternberg & Butcher, 1965) - first data on liver tumors dynamic contrast enhancement were published in 1980 by T. Araki (Araki et al., 1980; Foley et al., 1983). The first results of CT perfusion of the liver appeared in the late 80’s - early 90’s of the XX century (Miles, 1991; Miles et al., 1993; Mathieu et al., 1986; Rees, 1992).

Along with the altered metabolic profile, liver tumors are characterized by pathological hemodynamics, which is reflected in CT perfusion. The activity of tumor growth is determined by its neoangiogenesis and involvement of existing vessels with their subsequent tumor transformation. Tumor blood vessels have irregular lumen diameter and abnormal branching character, their basal membrane is fragmented (Dudarev & Akifiev, 1997; Prokop et al., 2003; Asayama et al., 2008). These specific criteria have an impact on diagnosis and treatment. For example, the absence of a basal membrane leads to a decrease in the tightness of the vessel, and then to an increase in interstitial pressure, which is reflected in the disruption of therapeutic agents transport and CA in tumor tissue (Tong et al., 2004).

There are signs of high vascularization of HCC nodes with a predominance of arterial perfusion (high values ​​of BF and ALP), while blood volume (BV) and portal blood flow (PVP) in them are reduced. Highly differentiated HCC has a low amount of mitosis, its cells are characterized by a highly organized membrane, high concentration of very-low-density lipoproteins, the vasculature and the parenchymal component are more structured. These features are reflected in the form of high values ​​of BF, BV and ALP, expressed by the accumulation of 18F–FCh.

Low-differentiated cancer is characterized by increased mitotic and enzymatic activity, less structured cell membrane, chaotic vascular architectonics, an increase in the stromal component, so the perfusion values of BF, BV and ALP are significantly lower than those for highly differentiated HCC. At the same time, the cell of the low-grade cancer, due to its enzymatic characteristics, intensively absorbs 18F–FDG (Castilla-Lièvre et al., 2016; Ippolito et al., 2010).

The results of our study showing that the definition of differentiation of HCC is possible by using PET/CT with 18F–FDG and 18F–FCh. Also it is possible to determine the vascularization of the tumor by using CT perfusion. The above results of our study do not contradict the data of other authors (Yamamoto et al., 2008; Paudya et al., 2008). However, our study is unique because of the use of PET/CT and CT perfusion in a single study («dual-time»).

In spite of the small number of patients, CT perfusion in combination with PET (18F–FDG and 18F–FCh) allowed to reveal the quantitative and visual correlation between the features of the metabolism and angiogenesis of hepatocellular carcinoma depending on the different degrees of its differentiation.

## Conclusions

The diagnostic capabilities of the complex of PET/CT techniques with 18F–FDG and 18F–FCh and CT perfusion in a single-scan mode for hepatocellular carcinoma were evaluated for the first time. The obtained data allow to assume that the integrated use of PET with 18F–FDG and 18F–FCh and CT perfusion in a single study improves the differential diagnostic possibilities of PET/CT diagnostics, which can find application in planning and prognosis of the disease. However, it is necessary to increase the number of patients to determine the prognostic value of the study.

## References

[CR1] Ackerstaff E., Glunde K., Bhujwalla Z M. Choline phospholipid metabolism: a target in cancer cells? J Cell Biochem. 2003; 90:3. р. 525-533 10.1002/jcb.10659 14523987

[CR2] Araki T. et al. Dynamic CT densitometry of hepatic tumors. Am J Roentgenol. 1980. 135:5. р. 1037-1043 10.2214/ajr.135.5.1037 6255782

[CR3] Argaud D. et al. Stimulation of glucose-6-phosphatase gene expression by glucose and fructose-2, 6-bisphosphate. J Biol Chem. 1997; 272:19. р. 12854-12861 10.1074/jbc.272.19.12854 9139747

[CR4] Asayama Y. et al. Arterial blood supply of hepatocellular carcinoma and histologic grading: radiologic-pathologic correlation. Am J Roentgenol. 2008; 190:1. р. 28-34 10.2214/AJR.07.2117 18094269

[CR5] Axel L. Cerebral blood flow determination by rapid-sequence computed tomography: theoretical analysis. Radiology. 1980; 137:3. р. 679-686 10.1148/radiology.137.3.7003648 7003648

[CR6] Bartolotta, T. V. et al. Focal liver lesions: contrast-enhanced ultrasound. Abdom Imaging. 2009; 34:2. р. 193-209 10.1007/s00261-008-9378-6 18317833

[CR7] Beasley R P. et al. Hepatocellular carcinoma and hepatitis B virus: a prospective study of 22 707 men in Taiwan. London: Lancet. 1981; 318: 8256. р. 1129–1133 10.1016/s0140-6736(81)90585-7 6118576

[CR8] Berteloot A., Vidal H., Van der Werve G. Rapid kinetics of liver microsomal glucose-6-phosphatase. Evidence for tight-coupling between glucose-6-phosphate transport and phosphohydrolase activity. J Biol Chem. 1991; 266:9. р. 5497-5507 1848552

[CR9] Bosch F X., Ribes J., Borràs J. Epidemiology of primary liver cancer. Semin Liver Dis. 1998; 19:3. р. 271-285 10.1055/s-2007-1007117 10518307

[CR10] Bray F (2012). Global cancer transitions according to the human development index (2008-2030): a population-based study. The lancet Oncology.

[CR11] Castilla-Lièvre M A. et al. Diagnostic value of combining 11C-choline and 18F-FDG PET/CT in hepatocellular carcinoma. Eur J Nucl Med Mol Imaging. 2016; 43:5. р. 852-859 10.1007/s00259-015-3241-0 26577938

[CR12] Chissov V.I. Oncology: NAT. Hands. Moscow: GEOTAR-media. 2008; р. 205–212

[CR13] Delbeke D. et al. Evaluation of benign vs malignant hepatic lesions with positron emission tomography. Arch Surg. 1998; 133:5. р. 510-516 10.1001/archsurg.133.5.510 9605913

[CR14] Dolgushin M. B., Pronin I. N., Fadeeva L. M. et al. SP SWAN (3.0 Tesla MRI) and CT perfusion in a comprehensive assessment of the structural features of brain metastases and malignant gliomas. Radiation diagnostics and therapy. 2012; 3. р. 41-51

[CR15] Dudarev V. S., Akifiev V. V. Modern interventional radiology. Radiation Diagnostics. 1997; 1. р. 26-27

[CR16] Ershov V. A. Morphological criteria of primary liver cancer. Medicine. 2009; 11:3. р. 204

[CR17] Foley W. D. et al. Contrast enhancement technique for dynamic hepatic computed tomographic scanning. Radiology. 1983; 147:3. р. 797-803 10.1148/radiology.147.3.6844616 6844616

[CR18] GLOBOCAN (2014) Estimated cancer incidence, mortality, and prevalence worldwide in 2012. IARC

[CR19] Highleyman L. Does differ Hepatocellular carcinoma in people with hepatitis B and C? 2015 ASCO annual meeting. 2015; р. 1

[CR20] Hwang K. H. et al. Evaluation of patients with hepatocellular carcinomas using [11 C] acetate and [18 F] FDG PET/CT: a preliminary study. Appl Radiat Isot. 2009; 67: 7. р. 1195-1198 10.1016/j.apradiso.2009.02.011 19342249

[CR21] Ippolito D. et al. Perfusion CT in cirrhotic patients with early stage hepatocellular carcinoma: assessment of tumor-related vascularization. Eur J Radiol. 2010; 73:1. р. 148-152 10.1016/j.ejrad.2008.10.014 19054640

[CR22] Karmazanovskii G. G., Szymanowski N. L. Diagnostic effectiveness of a new magnetic resonance contrast agents “Primovist” (gadaxtoma acid) in identifying primary and secondary liver tumors. Med Visualization. 2007; 6. р. 135-143

[CR23] Kety S. S., Schmidt C. F. The nitrous oxide method for the quantitative determination of cerebral blood flow in man: theory, procedure and normal values. J Clin Investig. 1948; 27:4. р. 476 10.1172/JCI101994 PMC439518 16695568

[CR24] Kuang Y. Positron emission tomography imaging of hepatocellular carcinoma with radiolabeled choline. Case Western Reserve University. Ann Arbor. 2009. р. 38–48

[CR25] Lukyanchenko AB, Medvedev BM (2004). Magnetic resonance imaging in the diagnosis and differential diagnosis of focal liver disease. Bulletin of RONC N N Blokhin of the RAMS.

[CR26] Mathieu D. et al. Hepatic adenomas and focal nodular hyperplasia: dynamic CT study. Radiology. 1986; 160:1. р. 53-58 10.1148/radiology.160.1.3520655 3520655

[CR27] Matsui O. et al. Benign and malignant nodules in cirrhotic livers: distinction based on blood supply. Radiology. 1991; 178:2. р. 493-497 10.1148/radiology.178.2.1846240 1846240

[CR28] Meier P., Zierler, K. L. On the theory of the indicator-dilution method for measurement of blood flow and volume. J Appl Physiol. 1954; 6:12. р. 731-744 10.1152/jappl.1954.6.12.731 13174454

[CR29] Miles K. A. Measurement of tissue perfusion by dynamic computed tomography //Br J Radiol. – 1991. – T. 64. . – P. 409-412 No. 761 10.1259/0007-1285-64-761-409 2036562

[CR30] Miles K. A., Haybal M. P., Dixon K. Functional images of hepatic perfusion obtained with dynamic CT. Radiology. 1993; 188:2. р. 405-411 10.1148/radiology.188.2.8327686 8327686

[CR31] Miller, K. D. et al. Global cancer epidemiology and the cancer divide. Global perspectives on cancer: incidence, care, and experience. California: Praeger. 2015; р. 5

[CR32] Mirk P. et al. Ultrasonographic patterns in hepatic hemangiomas. Journal of clinical ultrasound: JCU. 1982; 10:8. р. 373 10.1002/jcu.1870100805 6816817

[CR33] Nordlie R. C., Foster J. D., Lange A. J. Regulation of glucose production by the liver. Annu Rev Nutr. 1999; 19:1. р. 379-406 10.1146/annurev.nutr.19.1.379 10448530

[CR34] Pantoja E. Angiography in liver hemangioma. Am J Roentgenol. 1968; 104:4. р. 874-879 10.2214/ajr.104.4.874 5725724

[CR35] Patyutko Yu I (2011). Hepatocellular liver cancer. Bulletin of medical iInternet conferences. Limited Liability Company Science and Innovations.

[CR36] Paudya B. et al. Clinicopathological presentation of varying 18F-FDG uptake and expression of glucose transporter 1 and hexokinase II in cases of hepatocellular carcinoma and cholangiocellular carcinoma. Ann Nucl Med. 2008; 22:1. р. 83-86 10.1007/s12149-007-0076-1 18250992

[CR37] Podymova SD (1998). Liver disease: a guide for physicians.

[CR38] Pritchard P. H., Vance D. E. Choline metabolism and phosphatidylcholine biosynthesis in cultured rat hepatocytes. Biochem J. 1981; 196. р. 261-267 10.1042/bj1960261 PMC1162990 6272753

[CR39] Prokop M., Galanski M., Van Der Molen A. J. Spiral and multislice computed tomography of the body. Thieme. 2003; р. 234-237

[CR40] Pronin I. N. et al. Perfusion CT: a study of cerebral hemodynamics in the norm. Medical imaging. 2007; 3. р. 8-12

[CR41] Rees S. Measurement of tissue perfusion by dynamic computed tomography. Br J Radiol. 1992; 65:774. р. 554-555 10.1259/0007-1285-65-774-554-b 1628194

[CR42] Salomao M., McMillen E., Lefkowitch, J. H. Recent advances in the classification of hepatocellular carcinoma. Diagnostic histopathology. 2012; 18:1. р. 37-45

[CR43] Soget SR (2013). Hepatocellular cancer (epidemiology, radiation diagnosis, modern aspects of treatment). Practical Medicine.

[CR44] Sukonko O G. Hepatocellular carcinoma. Algorithm for the diagnosis and treatment of malignant tumors. Minsk:Medicina. 2012; р. 12–14

[CR45] Talbot J. N. et al. Detection of hepatocellular carcinoma with PET/CT: a prospective comparison of 18F-fluorocholine and 18F-FDG in patients with cirrhosis or chronic liver disease. J Nucl Med. 2010; 51:11. р. 1699-1706 10.2967/jnumed.110.075507 20956466

[CR46] Ternberg J. L., Butcher H. R. Blood-flow relation between hepatic artery and portal vein. Science. 1965; 150:3699. р. 1030-1031 10.1126/science.150.3699.1030 5843615

[CR47] Ternovoy SK, Shiganova SV (1999). Magnetic resonance imaging in the diagnosis of focal liver diseases (literature review).

[CR48] Tiferes D. A, D’ippolito G. Liver neoplasms: imaging characterization. Radiol Bras. 2008; 41:2. р. 119-127

[CR49] Tong R. T. et al. Vascular normalization by vascular endothelial growth factor receptor 2 blockade induces a pressure gradient across the vasculature and improves drug penetration in tumors. Cancer Res. 2004; 64:11. р. 3731-3736 10.1158/0008-5472.CAN-04-0074 15172975

[CR50] Tulin, P. E., Dolgushin M. B., Patyutko Yu. I. et al. PET/CT with 18F-FDG and 18F-choline in the diagnosis of mixed hepatocholangiocarcinoma cancer. Clinical observation Diagnostic and interventional radiology.2015; 9:1. р. 91-99

[CR51] Yamamoto Y. et al. Detection of hepatocellular carcinoma using 11C-choline PET: comparison with 18F-FDG PET. J Nucl Med. 2008; 49:8. р. 1245-1248 10.2967/jnumed.108.052639 18632827

[CR52] Zajko A. B. et al. Angiography of liver transplantation patients. Radiology. 1985; 157:2. р. 305-311 10.1148/radiology.157.2.3901102 PMC2962557 3901102

[CR53] Zeisel S. H., Blusztajn, J. K. Choline and human nutrition. Annu Rev Nutr. 1994; 14:1. р. 269-296 10.1146/annurev.nu.14.070194.001413 7946521

